# Enteroendocrine cell expression of split-GAL4 drivers bearing regulatory sequences associated with panneuronally expressed genes in *Drosophila melanogaster*

**DOI:** 10.17912/micropub.biology.000628

**Published:** 2022-08-19

**Authors:** Jessica M. Holsopple, Kevin R. Cook, Ellen M. Popodi

**Affiliations:** 1 Bloomington Drosophila Stock Center, Department of Biology, Indiana University

## Abstract

In *Drosophila melanogaster* , hormone-secreting enteroendocrine cells are important for communication from the midgut to other tissues. Many lexA, GAL4, and split-GAL4 drivers that direct gene expression in enteroendocrine cells also confer expression in hormone-secreting cells of the central nervous system. This study examines the midgut expression of selected lexA, GAL4, and split-GAL4 transgenes carrying enhancer fragments previously associated with panneuronal gene expression to assess the experimental usefulness of these drivers for distinguishing the endocrine influences of CNS versus midgut cells on physiological processes.

**Figure 1.**
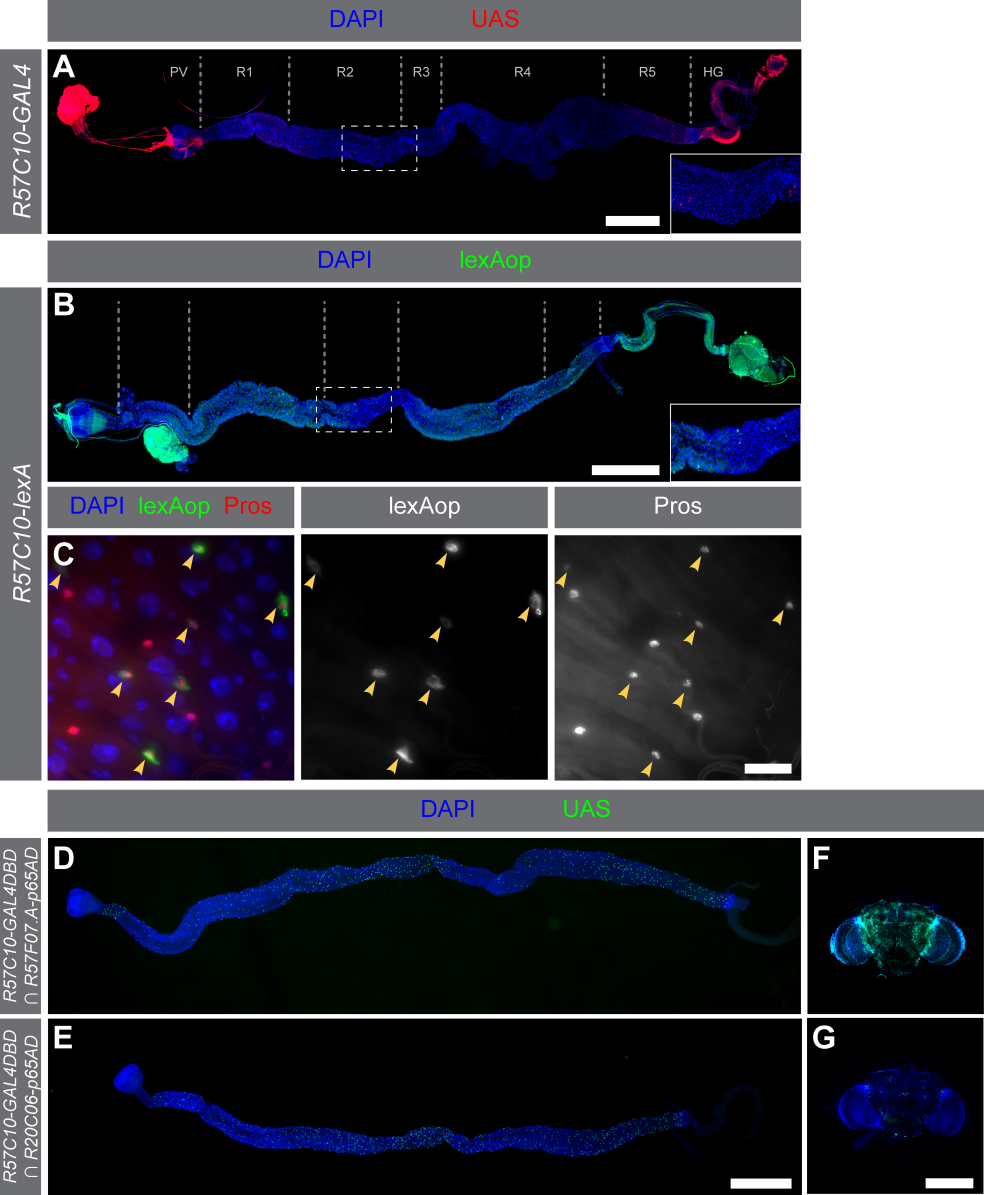
(A) *R57C10-GAL4* drives reporter expression in a small population of cells scattered throughout the adult midgut. *10XUAS-IVS-mCD8::RFP* reporter expression is shown superimposed on nuclear DAPI staining. The primary midgut regions (Buchon et al. 2013) are labeled above the gut. Scale bar 500 µM. The inset shows an enlargement of the indicated region of the central midgut. (B) *R57C10-lexA* drives reporter expression in a small population of cells scattered throughout the adult midgut. *13XLexAop2-mCD8::GFP* reporter expression is shown superimposed on nuclear DAPI staining. The primary midgut regions (also labeled in (A)) are delineated above the micrograph of the gut. Scale bar 500 µM. The inset shows an enlargement of the indicated region. (C) The cells detected by *R57C10-lexA* are positive for anti-Prospero (Pros) antibody, indicating that they are EEs (arrows). *13XLexAop2-mCD8::GFP* reporter expression is shown in the center column and anti-Pros staining is shown in the right column. The fluorescence patterns are superimposed on nuclear DAPI staining in the left column. Images are taken from R3 of the adult midgut. Scale bar 20 µM. (D–G) *R57C10-GAL4DBD* drives expression in both midguts (D, E) and brains (F, G) when combined with EE reference drivers. *UAS-Stinger* expression is shown superimposed on nuclear DAPI staining. (D, E) *R57C10-GAL4DBD* drives expression in many EEs. Midgut patterns are similar with both EE reference drivers, although *R20C06* drivers detect more EEs than *R57F07* drivers. Scale bar 500 µM. (D–G). (F, G) In contrast, there are distinct differences in brain expression patterns depending on the EE reference driver. The *R57F07* driver (F) confers considerably more brain expression than the *R20C06* driver (G). Scale bar 300 µM. Genotypes: (A) y ^1^ w ^*^ P{10XUAS-IVS-mCD8::RFP}attP18 P{13XLexAop2-mCD8::GFP}su(Hw)attP8/w ^1118^ ; P{GMR57C10-GAL4}attP2/+ (B,C) y ^1^ w ^*^ P{10XUAS-IVS-mCD8::RFP}attP18 P{13XLexAop2-mCD8::GFP}su(Hw)attP8/w ^1118^ ; P{GMR57C10-lexA}attP40/+ , (D, F) *w ^1118^ ; P{R20C06-p65.AD}attP40/+; P{R57C10-GAL4.DBD}attP2/P{UAS-Stinger}3* (E, G) *w ^1118^ ; P{R57F07.A-p65.AD}attP40/+; P{R57C10-GAL4.DBD}attP2/P{UAS-Stinger}3* .

## Description

In *Drosophila melanogaster* , many peptide hormones are secreted by both central nervous system cells and midgut enteroendocrine cells (EEs) (Wegener and Veenstra 2015). To understand endocrine communication, investigators need experimental tools to manipulate gene expression in each of these cell types independently. Unfortunately, most relevant lexA, GAL4, and split-GAL4 drivers that have been characterized to date direct expression in both cell types.

Kubrak et al *.* (2022) designed an effective approach for limiting GAL4 activity to EEs and eliminating it in the brain. They used an enhancer fragment ( *R57C10* ) associated with the *nSyb* gene to express GAL80 panneuronally, and thereby suppress GAL4 transcriptional activation of a *UAS* construct in the brain. Specifically, *R57C10-GAL80* prevented *AstC-GAL4* from activating a *UAS-RNAi* construct directed against mRNA expressed by the *AstC* peptide-hormone gene in the brain. Consequently, *AstC* transcript levels were strongly reduced in the midgut, but normal in the brain.

These results suggest that the *R57C10* enhancer fragment directs expression only in the brain. Indeed, Kubrak et al. (2022) did not see fluorescence in midgut cells in flies carrying both *R57C10-GAL4* and *UAS-GFP* . Concurrently, we characterized driver expression directed by this enhancer fragment as a follow up to our previous work (Ariyapala et al. 2020). When we mated flies carrying *R57C10-GAL4* and *R57C10-lexA* drivers individually to flies carrying the two reporters *10XUAS-IVS-mCD8::RFP* and *13XLexAop2-mCD8::GFP* , we saw fluorescence in a small number of midgut cells in adult female progeny in midgut regions 1A, 2C, 3, 4A, and 5A and B (Figure 1A and B). The lexA driver directed midgut expression in a few more cells than the GAL4 driver. As expected, we found that all fluorescent midgut cells were EEs, as they stained with anti-Prospero (Pros) antibody (Figure 1C). The apparent contradiction between our results and those of Kubrak et al. (2022) is likely explained by the use of different *UAS* reporters. We used an enhanced reporter ( *10XUAS-IVS-mCD8::RFP* ) while they used a less sensitive reporter ( *UAS-mCD8::GFP* ) (Kim Rewitz, personal communication). Despite evidence that *R57C10* promotes low-level gene expression in EEs, Kubrak et al. (2022) saw little EE expression of *AstC* mRNA in flies carrying the *R57C10-GAL80* , *AstC-GAL4* , and *UAS-AstC-RNAi* constructs and observed the same alterations in gut functions that were induced when *AstC* expression was knocked down in flies carrying the *UAS-AstC-RNAi* construct and an EE-specific GAL4 driver. These results indicate that any inhibition of GAL4 activity that may have come from *R57C10-GAL80* expression in EEs was not sufficient to allow physiologically significant *AstC* expression, and that, with appropriate controls, the *R57C10-GAL80* construct may prove useful in similar experiments to limit the expression of other peptide hormones to the brain.

To evaluate the utility of other drivers for manipulating midgut gene expression, we examined the expression of eleven split-GAL4 drivers made by Dionne et al. (2018) with enhancer fragments originating within 10 kb of genes frequently used as panneuronal markers ( *R27E08* and *R71C07* from *elav* , *R23E05* from *fne* , and *R14A01* , *R57C10* , *VT024098* , and *VT024099* from *nSyb* ). We combined each split-GAL4 driver with a *UAS-Stinger* reporter and previously established split-GAL4 EE reference drivers that use the enhancer fragments *R57F07* or *R20C06* (Ariyapala et al. 2020; Holsopple et al. 2022). Interestingly, *R57C10-p65AD* and *-GAL4DBD* , which carry the same enhancer fragment as the lexA and GAL4 drivers characterized above, drove expression in many EEs in all midgut regions when combined with both reference drivers (Figure 1D and E)—patterns that were different than the restricted expression seen with the *R57C10* GAL4 and lexA drivers. We have seen discrepancies between the expression patterns of GAL4 and split-GAL4 drivers bearing the same enhancer fragments in the past and they appear to be common (e.g. Ariyapala et al. 2020 and Holsopple et al. 2022). *R27E08-p65AD* also directed widespread midgut expression, while the *VT024098-p65AD* and *-GAL4DBD* drivers directed expression in only small numbers of cells limited to the middle midgut. We saw no EE expression with the remaining drivers: *R71C07-p65AD* and *-GAL4DBD* , *R14A01* - *GAL4DBD* , *R23E05-p65AD* and *-GAL4DBD* , and *VT024099-GAL4DBD* . We also screened two split-GAL4 drivers generated by Luan et al. (2006) that carry *elav* regulatory sequences. *elav-GAL4DBD* directed expression in EEs in all midgut regions, while *elav-GAL4AD* directed expression in only a few cells in the middle midgut. Consistent with the findings of Holsopple et al. (2022), there were notable differences in expression patterns based on the EE reference driver used (Figure 1D and E). For four experimental drivers, the *R20C06* reference driver directed expression in more midgut cells than the *R57F07* driver. *VT024098-p65AD* was the only driver with which a *R57F07* reference driver directed expression in more cells. The midgut expression patterns are documented in detail in the Extended Data table and at https://bdsc.indiana.edu/stocks/gal4/midgut_EEs.html .

We saw brain expression in all driver combinations with midgut expression except *elav-GAL4AD* combined with *R20C06-GAL4DBD* . Because Luan et al. (2006) described *elav-GAL4AD* as having panneuronal expression, we were surprised to see no brain expression. This driver also showed unexpected midgut expression patterns in combination with the EE reference drivers above. It is possible that *elav-GAL4AD* is not actually expressed in all CNS cells or widely in the midgut, or that its activation domain does not promote *UAS-GFP* expression as strongly as the activation domain in the other constructs, but it seems more likely that these are cases of idiosyncratic interaction between split-GAL4 drivers—as seen frequently by Ariyapala et al. (2020) and Holsopple et al. (2022). Regardless, we know of no other driver combination activating *UAS* expression in EEs but not the CNS. Unfortunately, *UAS* activation is confined to a small subset of EEs, so the combination may have limited experimental utility. We do not know the significance of this EE subset in terms of midgut physiology.

The drivers without midgut expression are potentially valuable for activating expression of *UAS* constructs in the brain. We saw seven driver combinations that directed expression in the brain but not the midgut. Five were combinations with the *R57F07* drivers ( *R71C07-p65AD* and *-GAL4DBD* , *R14A01-GAL4DBD* , *R23E05-GAL4DBD* , and *VT024099-GAL4DBD* ) and two were combinations with *R20C06* drivers ( *R71C07-p65AD* and *VT024099-GAL4DBD* ). These driver pairs are particularly interesting because they should allow researchers to manipulate CNS cells while leaving midgut cells unchanged. The remaining five driver combinations directed no detectable midgut or brain expression.

Among the driver combinations directing *UAS* reporter expression in the brain, we see considerable heterogeneity in expression patterns. In contrast to the midgut and consistent with the results of Holsopple et al. (2022), the *R20C06* reference drivers conferred less reporter expression in the brain than the *R57F07* drivers (e.g. Figure 1F and G). It will take considerably more work to identify the cell populations and evaluate the relationships between *UAS* marker expression and peptide-hormone secretion. Our preliminary characterizations of brain expression patterns for these driver combinations are also documented at https://bdsc.indiana.edu/stocks/gal4/midgut_EEs.html .

Further work is necessary to identify split-GAL4 drivers better suited to directing *UAS* construct expression exclusively in EEs or peptide hormone-secreting CNS cells, but we hope the results of this study will provide avenues for continued research.

## Methods

Whole guts and brains were collected from 5-to-8 day old adult female flies and processed, stained, scored, and documented as described in Ariyapala et al *.* (2020) and Holsopple et al *.* (2022).

## Reagents

**Table:** 

Stock Number	Bloomington Drosophila Stock Center genotype
23867	w ^*^ ; P{w ^+mC^ =UAS-2xEGFP}AH2; P{w ^+mC^ =elav-GAL4.DBD}H4A1
23868	w ^*^ ; P{w ^+mC^ =UAS-2xEGFP}AH2; P{w ^+mC^ =elav-GAL4.AD}I1A1
32229	y ^1^ w ^*^ P{y ^+t7.7^ w ^+mC^ =10XUAS-IVS-mCD8::RFP}attP18 P{y ^+t7.7^ w ^+mC^ =13XLexAop2-mCD8::GFP}su(Hw)attP8
39171	* w ^1118^ ; P{y ^+t7.7^ w ^+mC^ =GMR57C10-GAL4}attP2 *
52817	* w ^1118^ ; P{y ^+t7.7^ w ^+mC^ =GMR57C10-lexA}attP40/CyO *
68517	* w ^1118^ ; P{y ^+t7.7^ w ^+mC^ =R71C07-GAL4.DBD}attP2 *
68606	* w ^1118^ ; P{y ^+t7.7^ w ^+mC^ =R71C07-p65.AD}attP40 *
68939	* w ^1118^ ; P{y ^+t7.7^ w ^+mC^ =R14A01-GAL4.DBD}attP2 *
69152	* w ^1118^ ; P{y ^+t7.7^ w ^+mC^ =R57C10-GAL4.DBD}attP2 *
69829	* w ^1118^ ; P{y ^+t7.7^ w ^+mC^ =R23E05-GAL4.DBD}attP2 *
69841	* w ^1118^ ; P{y ^+t7.7^ w ^+mC^ =R20C06-GAL4.DBD}attP2 *
70048	* w ^1118^ ; P{y ^+t7.7^ w ^+mC^ =R27E08-p65.AD}attP40 *
70585	* w ^1118^ ; P{y ^+t7.7^ w ^+mC^ =R20C06-p65.AD}attP40; MKRS/TM6B, Tb ^1^*
70601	* w ^1118^ ; P{y ^+t7.7^ w ^+mC^ =R23E05-p65.AD}attP40; MKRS/TM6B, Tb ^1^*
70746	* w ^1118^ ; P{y ^+t7.7^ w ^+mC^ =R57C10-p65.AD}attP40; MKRS/TM6B, Tb ^1^*
72297	* w ^1118^ ; P{y ^+t7.7^ w ^+mC^ =VT024098-p65.AD}attP40 *
72656	* w ^1118^ ; P{y ^+t7.7^ w ^+mC^ =VT024098-GAL4.DBD}attP2 *
73983	* w ^1118^ ; P{y ^+t7.7^ w ^+mC^ =VT024099-GAL4.DBD}attP2 *
84277	* w ^1118^ ; P{w ^+mC^ =UAS-Stinger}2 *
84278	y ^1^ w ^*^ ; wg ^Sp-1^ /CyO; P{w ^+mC^ =UAS-Stinger}3
91402	* w[; P{y ^+t7.7^ w ^+mC^ =R57F07-p65.AD.A}attP40; P{y ^+t7.7^ w ^+mC^ =UAS-DSCP-6XEGFP}attP2 *
91403	w ^*^ ; PBac{y ^+mDint2^ w ^+mC^ =UAS-DSCP-6XEGFP}VK00018; P{y ^+t7.7^ w ^+mC^ =R57F07-GAL4.DBD.A}attP2/TM6C, Sb ^1^ Tb ^1^

**Table:** 

**Antibody**	**Available from**
Mouse anti-Prospero monoclonal (MR1A)	Developmental Studies Hybridoma Bank
Goat Alexa Fluor 568-conjugated anti-mouse (A-11031)	Thermo Fisher Scientific

## Extended Data

Description: This table provides detailed midgut and brain scoring data for each split-GAL4 pair examined in this study. Resource Type: Dataset. DOI: 10.22002/D1.20271
